# Coinfection between SARS‐CoV‐2 and other respiratory tract viruses

**DOI:** 10.1002/jcla.24365

**Published:** 2022-04-04

**Authors:** Öner Özdemir, Ümmügülsüm Dikici

**Affiliations:** ^1^ 52992 Division of Allergy and Immunology Department of Pediatrics Faculty of Medicine Research and Training Hospital of Sakarya University Sakarya University Sakarya Turkey

**Keywords:** Coinfection, COVID‐19, respiratory tract viruses, SARS‐CoV‐2

## CONFLICT OF INTEREST

The authors declare that they have no conflict of interest.

## AUTHORS CONTRIBUTIONS

Dr. Özdemir wrote the article and Dr. Dikici assisted to Dr. Özdemir.

Dear Editor,

We have read the article titled to ‘Coinfection with severe acute respiratory syndrome coronavirus‐2 and other respiratory viruses at a tertiary hospital in Korea’ by Lee et al^1^ In this manuscript, we agree with the authors that SARS‐CoV‐2 coinfections with other respiratory tract infection (RTI) viruses recently have begun to draw attention.^2^, ^3^ In this study, the authors explored specific RTI viruses and their possible relations with SARS‐CoV‐2.

First, what we wondered in this interesting study is that could especially other coronaviruses (human coronavirus 229E x2, human coronavirus OC43) be a cross‐reaction, associated with testing method?

Secondly, also, as a clinician, was there any effect on clinical picture of the patients due to these coinfections of SARS‐CoV‐2? Any change in markers of acute phase of infection/inflammation and/or clinical findings was detected due to these coinfections? Could these coinfections be clinically irrelevant or insignificant?

Furthermore, we want to mention of our patient has suffered SARS‐CoV‐2 coinfection with parainfluenza 4 virus. In this study,^1^ the authors did not come across this RTI virus, like human metapneumovirus and respiratory syncytial virus (RSV).^4^, ^5^, ^6^ Also, in a retrospective study, the most common coinfected microorganism was found to be *Mycoplasma pneumoniae* (25%), followed by virus (7%) and bacteria (5%).^7^ Similar to previous literature,^2^, ^3^, ^4^, ^5^, ^6^ the authors demonstrated four times human rhinovirus (A and B), thrice both human coronavirus (229E and OC43) and mastadenovirus A, and once Influenza A virus.^1^

Our patient is 2.1/2‐year‐old male patient who was followed up with the diagnosis of severe form of transient hypogammaglobulinemia of infancy and received IVIG treatment once every 4 weeks. He was admitted with fever, cough, and anorexia lasting for the last 3 days. His physical examination revealed bilateral ronchi and localized subcrepitant rales on the lungs. Hemogram showed WBC: 19.230/mm^3^, CRP: 55 mg/L, ESR: 59 mm/hr, procalcitonin: 0.73 µg/L (*n*:<0.5), Ferritin: 93.5 ng/ml, LDH: 724 U/L, AST: 74 U/L, ALT: 22 U/L, PT: 11.5’’, aPTT: 30.5’’, and D‐dimer: 277 µ/L. The respiratory viral panel, including 13 species of RTI viruses, obtained from the patient showed positivity for SARS‐CoV‐2 and parainfluenza 4 virus. Ceftriaxone (100 mg/kg/day) was started in the patient due to ongoing fever, cough, fatigue, and elevated acute phase reactants. Five doses of IVIG at the dose of 0.4 g/kg for 5 days were given. On the 7th day of hospitalization, clinical symptoms of the patient were improved and discharged without any complaints.

In very near future, SARS‐CoV‐2 coinfections with other RTI viruses and other bacterial and fungal microorganisms in human will be discussed more and gain importance to take care of these patients.

## INFORMED CONSENTS

All patients or their relatives had to sign the informed consent before their data were recruited.

## Data Availability

The data that support the findings of this study are available on request from the corresponding author.
